# Geospatial approach to pluvial flood-risk and vulnerability assessment in Sunyani Municipality

**DOI:** 10.1016/j.heliyon.2024.e38013

**Published:** 2024-09-18

**Authors:** Aaron Tettey Tetteh, Abdul-Wadood Moomen, Lily Lisa Yevugah, Albert Tengnibuor

**Affiliations:** a School of Mines and Built Environment, University of Energy and Natural Resources, Sunyani, Ghana; b Department of Geospatial Sciences, School of Geosciences, University of Energy and Natural Resources, Ghana

**Keywords:** Flood-prone, Sunyani municipality, Precipitation, Drainage density, Geospatial techniques, AHP, Vulnerability assessment

## Abstract

Historically, and in recent times, efforts have been to understand, predict, analyze, and quantify floods and their impacts in various countries of the globe. Although recent scientific advances have introduced approaches to assessing the risks presented by flooding, little studies have been carried out in the Sunyani Municipality of Ghana for generating a pluvial flood-risk and vulnerability map for risk identification, resilience, emergency preparedness, and urban spatial planning. In this study, five parameters that influence both pluvial and fluvial flooding were assessed to map flood-prone areas within the Sunyani Municipality. These are precipitation, drainage density, LULC, elevation, and slope, which were integrated in GIS. Using an AHP, weights were assigned to each parameter based on its level of influence on flooding. The findings reveal that 21.32 % of the Sunyani Municipality lies within a highly flood-prone area, 39.65 % in a flood-prone area, while 28.06 % and 10.97 % in slightly flood-prone and not flood-prone areas respectively. Built-up areas close to watersheds with lower elevations and larger drainage density are the places that are highly flood-prone. Some towns within the highly flood-prone and flood-prone areas are Abesim, Newtown, Nkwarbeng, Baakoniaba, Kootokrom, and Penkwase. Highly valued infrastructure such as schools, churches, and hospitals have also been found within these highly flood-prone areas. These findings can aid the government and relevant stakeholders in disaster risk management to be better informed, and to effectively plan and prevent flood challenges in the Sunyani Municipality. Moreover, urban spatial planners in the study setting can consider incorporating the flood hazard maps generated from this study into their spatial plans for proactive physical developments.

## Highlights

• Five key parameters influencing flooding were assessed to map flood-prone areas in the Sunyani Municipality.
• Sentinel-2, STRM DEM, and CHIRPS data were used for flood analysis and map generation using ArcGIS.
• Geospatial analysis weighted precipitation, drainage density, LULC, slope, and elevation using AHP.
• Abesim, Newtown, Penkwase, and Kootokrom are among the areas prone to flooding, with flood-prone zones covering 39.65% of the study area.
• Maps can guide MMDAs in flood management and land-use planning to mitigate flood hazards in urban areas.

## Graphical abstract

**Figure:**
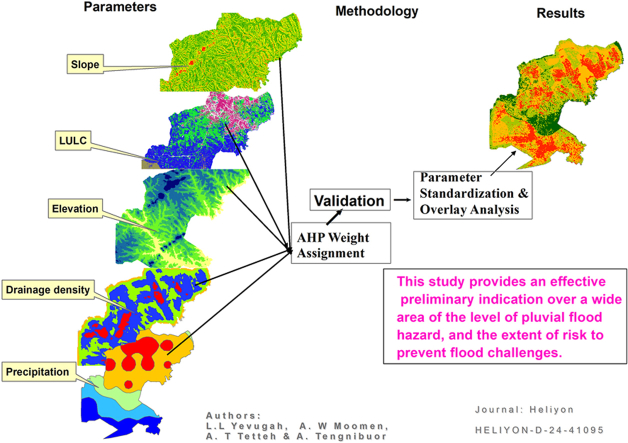

## 1 Introduction

Flooding is a global phenomenon, stands as the most prevalent natural disaster, and leads to both significant short and long-term economic, environmental, physical, and social losses [1]. Flooding accounts for 49 % of all disaster events and 44 % of all global natural disaster related fatalities in the 21st century, [2,3]. It is the excess flow that inundates the carrying capacity of the conveying or holding medium under prevailing conditions [4]. Flooding is projected to be exacerbated in the coming years, which could be attributed to many biophysical and anthropogenic factors including a combined effect of population growth, associated urbanization, and climate change [5]. Generally, flooding is classified into pluvial and fluvial processes. The former represents excessive surface runoff and rapid increase in water levels that exceed the carrying capacity of natural and engineered drainage systems, which is a function of intensive rainfall [6]. Examples include the 2004, and 2006 floods in Heywood, Greater Manchester, UK, the Swedish city of Malmö, and Port Harcourt Metropolis, Nigeria [[7], [8], [9]], which exposed victims’ unpreparedness due to lack of information. The latter represents overflow of stream or river channels and banks resulting from an increase in the water levels, which is also either a combined function of underground discharge and heavy rainfalls or one of them [10].

Although both flooding classes are not new phenomena, globally, the intensity and frequency with which they are occurring, particularly, are alarming and insipient to new urbanizing cities in Ghana. More recent flooding hazards across many cities in the country have generated a high level of awareness of the urgent need to more fully understand the nature of pluvial flooding and address the apparently increasing risks attendant. For instance, the Ashanti region of Ghana experienced the tragic loss of about 12 children due to flooding, while 29 individuals in the Upper East region succumbed to similar incidents in 2019 alone [11]. In June 2015, pluvial flooding in Accra affected around 53,000 people and caused an estimated US$100 million in economic damages [12]. More recently, the Akosombo dam spillage displaced 31,000 people across eight communities along the Volta basin, with Mepe being the most affected as of October 19, 2023 (Media report). As urban areas expand, drainage systems often fail to accommodate the increased runoff, exemplified by the severe flooding in the North Tongu constituency during the Akosombo dam spillage in 2023. To this end, the EU Green Paper on *Adapting to Climate Change* recommends that as a matter of building resilience and hazard mitigation, state agencies responsible for disaster risk management should conduct a national inventory of all flood risks, including pluvial and fluvial flood risks [13].

The Green Paper further recommends the need for partnership between state agencies and other institutions for developing tools and techniques towards mapping and modelling flooding risks. Mapping and modelling of the potential for pluvial flooding could provide sufficient information for a preliminary assessment of potential hazards. As such, this could help to develop a resource efficient strategy for subsequent mapping of areas susceptible to pluvial flooding, building warning systems and land use planning [14]. Although there have been recent studies that assess the risks of pluvial flooding in Ghana, due to existing data gaps, there are challenges in enhancing the resilience of cities to the impacts of pluvial flooding in Ghana. Accurately mapping spatial inundation areas of flooding in urban areas in the country is challenging. Moreover, there is little knowledge on the current vulnerability of cities that do not have well developed and comprehensive pluvial flood management plans for supporting urban resilience practices in Ghana. Although pluvial flooding is advancing in Ghana in many less developed cities like Sunyani, studies for identifying locations most susceptible to pluvial flooding within these cities are lacking. This form of study could generate data and provide responding organizations with more reliable warning information on possible flooding hazards than is currently available.

Some studies employ traditional flood monitoring that relies heavily on in-situ observations, which does not supply real-time comprehensive data across large regions, yet exposing on-site researchers to risks. Other studies resort to developing flood models whose performance exhibits significant variability when applied in urban areas due to the complexity of the terrain, the approach, and the parameters used in modelling [15].

Also, some studies use flood causative factors such as runoff, soil type, surface slope, surface roughness, drainage density, proximity to streams, land use and land cover (LULC), precipitation, normalized difference vegetation index (NDVI), flow accumulation, to generate flood susceptibility map [16,17]. In the Sunyani municipality, Wanyor & Morkla [18] applied existing elevation and hydrology data together with LULC maps to examine flood risk. However, their study has not outlined a rigorous methodology and geospatial tools employed for data analysis. In the Sunyani municipality, there are limited studies on mapping flood-prone zones, serving as the baseline for urban planners to make informed decisions about physical development planning. Also, previous studies do not employ a comprehensive integration of remotely sensed (RS) data such as precipitation, digital elevation model (DEM), and sentinel-2 images in a geographic information system (GIS) to map flood-prone areas in Sunyani. No recent studies focusing on Sunyani have attempted to generate data for warnings, to increase coverage, reliability, and accuracy, and to ensure that local authorities and affected areas have appropriate information, and sufficient time, to take effective action. The question is whether there is an existing and most up-to-date flood hazard risk map, and management plan based on reliable information for Sunyani, Ghana. Therefore, to address the aforementioned gaps, this study aims to map and assess pluvial flood-risk and associated potentially vulnerable areas within the Sunyani East Municipality based on Remote Sensing (RS), Geographic Information Systems (GIS), and Analytical Hierarchy Process (AHP). To achieve this aim, the specific objectives of this study are; (a) to detect the evolution and levels of pluvial flood occurrences in the study area; (b) to assess the extents of prevailing biophysical factors influencing flooding in the study area, (c) to understand the significance level of each biophysical factor influencing flooding in the study area, and (d) to map the spatial distribution of flood-prone areas within the municipality, given the presence and significance of each of these influencing factors to pluvial flooding in the study area. The findings of the study will significantly enhance pluvial flood monitoring, risks, and potential hazards, and provide technical support for spatial planning decisions.

## 2 Materials and methods

### 2.1 Study area

**Fig. 1:**
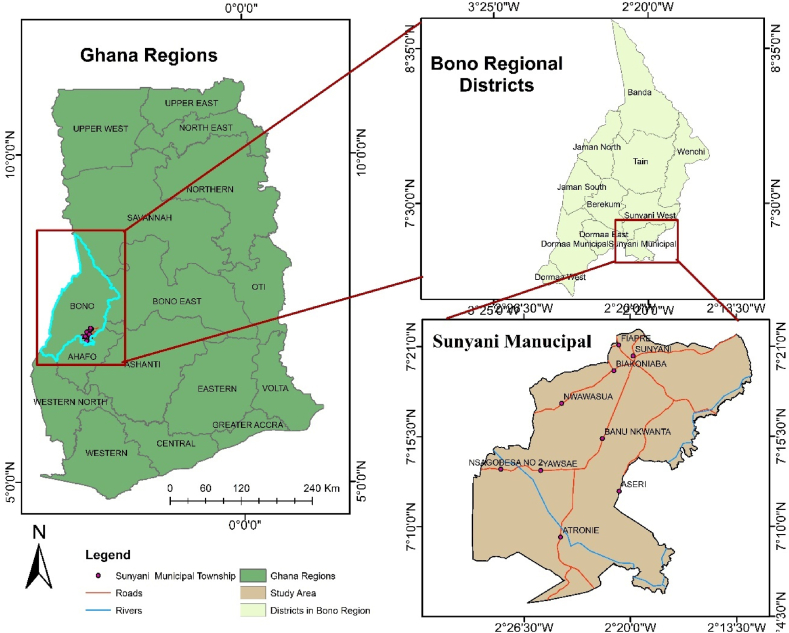
Map of the study area.

The Sunyani East Municipality is the administrative capital of Ghana's Bono region, located between latitudes 7°20′N and 7°05′N and longitudes 2°30′W and 2°10′W (Fig. 1). The Sunyani East Municipality has a population of 193,595, growing at an annual rate of 4.3 %. The population density stands at 382.9 individuals km^−2^ across a total area of 829.3 km^2^ [19]. As a result, the municipality has transitioned from an agriculture-based economy to service dominance and business centre, thereby becoming a key market hub. It is, however, estimated that approximately one-third of the municipality's arable land area is still available for current agricultural investments [20]. The municipality's economic activities are varied, with agriculture still playing a significant role. Major crops include maize, cassava, yam, vegetables, cocoa, and citrus fruits. The agricultural sector employs 45.9 % of the workforce, while the industrial sector (14.7 %) encompasses carpentry, bricklaying, timber industries, and construction [19].

Sunyani East Municipality falls within the wet semi-equatorial climatic regime, experiencing temperatures between 23 °C and 33 °C, high humidity, and annual rainfall of 88.987 cm. This climate allows for two farming seasons, although deforestation has led to a reduction in rainfall. The relatively flat terrain is favorable for large-scale agriculture but results in water shortages during dry periods. The area is characterized by Precambrian geological formations with mineral deposits and fertile ochrosols, supporting crops such as plantain, cocoyam, maize, cassava, and cocoa [21]. The major environmental challenges are deforestation, bushfires, and rapid urbanization and its associated environmental threats. The municipality's infrastructure and transportation networks enhance connectivity and trade. Additionally, open spaces and parks contribute to residents' quality of life and support environmental conservation and recreational activities.

### 2.2 Literature review

Flood risk mapping is an important component of disaster risk management worldwide. Hence, many approaches have been employed globally to analyze flood vulnerability [22]. The initial and crucial step in the process of mapping flood susceptibility is the creation of a flood inventory map. The main methods for acquiring a reliable and precise map involve extensively analyzing historical records and interpreting aerial photography [[23], [24], [25]]. Machine learning technique is one of the applied methods for flood vulnerability mapping. This is because of its ability to handle complex inputs has advanced pattern recognition, broadening its applications [24], [25]. The AHP-GIS model has been found to produce highly accurate flood maps with historical flood data and maximum causative factors of flooding. The combination of GIS and AHP is a robust technique for evaluating flood risk areas since it considers numerous flood parameters and assigns weights to the parameters influencing flooding [25]. Shah & Ai [26], study employed FR and AHP models to identify the flood susceptible areas in three districts Ghotki, Dadu, and Sanghar of Pakistan. Wahba et al. [27] assess and predict Flash Flood Hazard Susceptibility Mapping using a Random Forest Regression Model and Geographic Information System (GIS) in the Japanese prefecture of Ibaraki. A study of Ghosh & Das [28] applied random forest (RF) and support vector machine (SVM) models for wetland conversion risk assessment in India. Flood hazard mapping approaches can be grouped into there: physically-based models, empirical models, and physical modelling [29,30]. Each approach leverages different types of data and methods, often depending on the specific flood risk being assessed. Detailed hydrological data, such as river network geometry and topography are suited for fluvial flood assessments. Hence, they are classified under the physically based models approach [31,32]. However, these models are often limited in their ability to replicate the intricate processes of pluvial flooding and are typically less effective in regions where comprehensive hydrological data is scarce [33,34]. Empirical models offer a different approach, utilizing numerical inputs to simulate real-world flood processes across one, two, or three dimensions [34]. These models frequently incorporate data from Earth Observation and Remote Sensing, processed using Geographic Information Systems (GIS), making them versatile for both pluvial and fluvial flood mapping [35]. Within empirical models, various methods are employed, including multi-criteria decision-making methods (MCDM) [36], statistical methods [[37], [38], [39], [40]], and Machine Learning (ML) and Artificial Intelligence (AI) approaches [[41], [42], [43]]. The final approach: physical modelling approaches involve conducting experiments to validate flood predictions, which, while accurate, are often resource-intensive and time-consuming, limiting their practical application in large-scale or real-time scenarios.

In Africa, various approaches were utilized to assess flood vulnerability. Studies have proven that Frequency ratio, and Information Value (IV) models are widely used methods in flood susceptibility assessment. The FR measures the relationship between conditioning factors and flood occurrence, where FR > 1 indicates higher flood probability and FR < 1 indicates lower. The Information Value (IV) model, a bivariate statistical method, objectively assesses flood susceptibility by evaluating the spatial relationship between conditioning factors and flood occurrence probability. Higher IV values indicate stronger associations with flood probability, where IV > 0 suggests higher flood likelihood and IV < 0 indicates a weaker relationship [25]. Nevertheless, FR and IV models assume independent factors, risking inaccuracy when factors are interrelated. Some studies resort to the traditional approach of field observations. The study by Dube [44] utilized field observations in the form of stakeholder interviews approach to map the impact of flood hazards on tourism in South Africa.

Ghana has experienced several flooding incidents over the past decades. To predict future occurrences of flooding in other parts of Ghana, various methods and approaches have been employed by researchers. Danso et al. [17] integrated flooding causative parameters in GIS and AHP to assign weight for mapping flood hazards in Sekondi-Takoradi Metropolis, Ghana. The Digital Elevation Model (DEM) was utilized for the generation of slope, elevation, flow direction, flow accumulation, stream networks, proximity to streams, and drainage density for most study cases in Ghana [45]. Rainfall (Precipitation) is known to be the major influencer of flooding, a study by Dacosta et al. [46] revealed that inappropriate drainage culverts at various chainages on the road also add up to flooded road cases in Ghana. Rainfall intensity, flow accumulation, soil, land cover, and slope, elevation using the overlay analysis in ArcGIS to assess flooding in Accra metropolis in Ghana in a study of Baaba et al. [47]. Another study by Dekongmen et al. [48] utilized DEM to assess drainage density and elevation patterns to assess flood vulnerability areas in Accra, Ghana.

According to Wanyor & Morkla [18], a flood zone map for Sunyani was created using digital elevation, hydrological data, and land use information. This has not elaborated a robust methodology, with the use of tools and software such as GIS and a stepwise procedure leading to the generation of the flood zone map. However, the Wanyor & Morkla [18] study lacks a rigorous methodology and does not detail the geospatial tools used for data analysis. In Sunyani municipality, there is limited research on mapping flood-prone areas, which is essential for urban planners to make informed decisions about physical development. Additionally, previous studies have not fully integrated remotely sensed (RS) data, such as precipitation, digital elevation models (DEM), and Sentinel-2 imagery, within a geographic information system (GIS) for mapping flood-prone zones in Sunyani. Recent studies have also not focused on generating data to improve warning systems, enhance coverage, reliability, and accuracy, or provide local authorities and affected communities with the necessary information and time to take effective action.

Although ML and AI have shown promise in enhancing predictive accuracy, they require extensive computational resources and complex data processing. Given the limitations and requirements of different modelling approaches, this study adopts an empirical approach, utilizing Remote Sensing (RS) data in GIS combined with the Analytical Hierarchy Process (AHP) for weight assignment, to create a flood hazard map that reflects the unique characteristics of the study area. Remote Sensing (RS) data and GIS are powerful tools for flood risk assessment, especially when combined with the Analytical Hierarchy Process (AHP) for weight assignment. Unlike ML algorithms that adjust variable importance automatically, AHP provides a structured process where expert judgment assigns weights to flood-influencing factors, making it ideal for regions with limited data or specific local conditions. For instances of biases in expert judgments, the accuracy criteria for AHP weights assignments are utilized for validation. In this urban flood risk study, RS data has been used in GIS to map flood-prone areas by analyzing land use, elevation, and proximity to water bodies. AHP enhances this process by systematically evaluating the importance of each factor, resulting in more accurate and context-specific flood hazard maps. While ML and AI are useful for handling complex data, the combination of RS, GIS, and AHP offers a strong alternative, particularly where expert knowledge and data integration are key. This method supports the need for adaptable and locally relevant flood risk management strategies to utilize a novel methodology in the study area of incorporating RS data in GIS with AHP for mapping flood-prone areas.

#### 2.2.1 Flood assessment modelling

Generally, one or a combination of three major approaches are used to generate a flood hazard map. These approaches include a) physically-based, b) empirical, and c) physical modelling [29,30]. It must, however, be emphasised that the use of a specific type of data is not exclusive to any one type of model [49].

Physically-based models are adopted for flood prediction and early warning systems. However, these models require large hydrological input data, such as river network geometry and bathymetry, and topographic data [29], [30], [32]. This makes the models relevant for fluvial flooding assessments only and are largely unable to replicate the actual physical processes of complex flows and pluvial floods [31], [33], [34]. Empirical models, which rely on numerical inputs to flow equations in 1-, 2-, and 3- dimensions, are generally used to simulate real processes of a flow and flood occurrence [34]. The models rely on hydrological, topographic, Digital Elevation Model (DEM), and geomorphology data. These data are generally obtained through Earth Observation and Remote Sensing, and processed using GIS [50]. Hence, physically-based and empirical models are usually combined, using data from Remote Sensing and Earth Observation, for both pluvial and fluvial floods mapping. Empirical models are categorized into; i) multi-criteria decision-making methods (MCDM) [36], ii) statistical methods which include the bivariate and multivariate models [[37], [38], [39], [40]], and iii) Machine Learning approach (ML) [41 43], and Artificial Intelligence (AI) [42]. Physical modelling approaches require the conduct of series of experiments to validate their prediction performance output, which is a challenge both in terms of resource availability and, time.

### 2.3 Data sets used

**Fig. 2:**
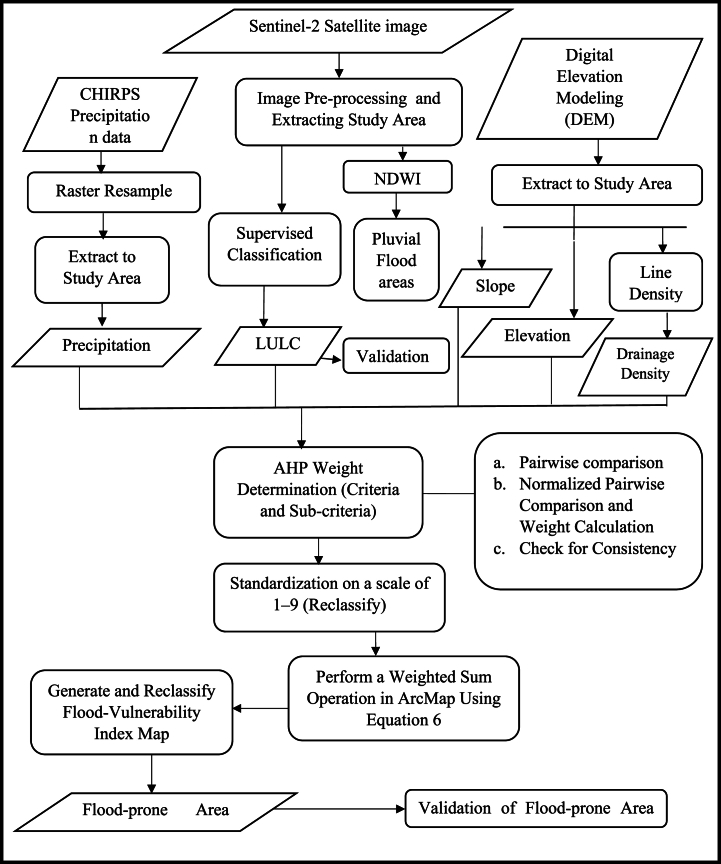
Flowchart of the methodology.

In this study, we use five different parameters to identify flood-prone areas in the Sunyani municipality. The diagnosis of various criteria depended on the maximum limitation method influencing flooding, which includes drainage density, population density, precipitation, elevation, slope, and land use/land cover (LULC) [1,51]. Table 1 below shows the list of the sources and types of data used for the flood-prone areas analysis. Fig. 2 is a flowchart, illustrating a comprehensive overview of the study's workflow.

**Table 1:** Data type and sources for flooding vulnerability assessment.

Indicator	Data type and year	Purpose and Technique
Land Use Land Cover (LULC)	Sentinel-2 Satellite images for 2016, 2018, 2020, 2022, and 2024, and Landsat 8 image of 2014.	supervised classification techniques to create maps of land use and land cover
Slope	STRM DEM 1-ARC. from USGS Earth Explorer, 2014. https://earthexplorer.usgs.gov/	Raster map of slope degree
Elevation	STRM DEM 1-ARC. from USGS Earth Explorer, 2014. https://earthexplorer.usgs.gov/	Raster map of elevation for different elevation classes
Drainage Density	STRM DEM 1-ARC. from USGS Earth Explorer, 2014 https://earthexplorer.usgs.gov/	Drainage density raster creation by (drainage length/sq. km)
Precipitation	Climate Hazards Group InfraRed Precipitation with Station data (CHIRPS), of 0.05 ^0^ resolution, 2023	Amount of rainfall in mm created from Precipitation

### 2.4 Parameters leading to flooding

Biophysical factors that lead to flooding considered in this study are Land Use and Land Cover Change (LULCC), precipitation, drainage density, elevation, and slope. Other influencing factors include population density and associated urbanization. This study considers LULC as an important parameter in mapping and modelling pluvial flood-prone areas in the study area. The land use and land cover change (LULCC) map was generated from Sentinel-2 satellite imagery obtained in 2014 and 2024. Historical data help to identify flood hazard potential areas, flood development, hazard intensity, flood depth, and spatial damage extents.

The precipitation (rainfall) patterns and drainage density have been reported as the major factors influencing pluvial flooding in major cities [52,53]. A study by Cobbinah et al. [53] show that precipitation has been reported as one of the vital causes of flooding in the Sunyani East Municipality. Increased rainfall in urban areas located on flat plains with poor drainage systems often results in pluvial flooding [35,54]. Hence, rainfall patterns have been analyzed in this study using satellite-based rainfall estimates. The average monthly rainfall data for 2023 was sourced from CHIRPS, known for its accurate rainfall forecasts and high spatial resolution of 0.05° when integrated with station data [55]. CHIRPS has been found to provide superior rainfall estimates in comparative studies [56]. Sulugodu and Deka [55] also demonstrated that CHIRPS data outperforms the Indian Meteorological Department (IMD) data in streamflow modelling. Additionally, Kibii and Plessis [57] utilized CHIRPS data to model the Pitman in South Africa, further showcasing its effectiveness. October was selected for this study because, along with September, it records the highest rainfall in the Sunyani East Municipality [58]. Areas with lower precipitation are less prone to direct experiences of pluvial flooding [[59], [60], [61]]. Additionally, the precipitation data from the CHIRPS were resampled to 10 m. This was done to ensure that the dataset has reduced cell size and enhanced spatial detail, and analytical precision when it was extracted to the study area covering a smaller portion of the world. A reclassification into 5 classes of the precipitation data was done.

Drainage density is the fraction of the total length of channels in a watershed on its total contributing area. Locations with higher drainage density are good indicators of high-flow accumulation path; making such locations more susceptible to flooding [16,17]. Hence, the drainage density of the study have been modelled using the DEM for the years 2004, 2014, and 2024. Drainage areas were computed using a single-direction flow algorithm, based on the steepest downslope neighbor of each pixel [62]. Slope gradient and elevation are other biophysical factors that intrinsically influence flooding [[63], [64], [65]]. Locations with lower slopes and lower elevation values are more susceptible to flooding than the reverse. Steep slopes and higher elevations are not vulnerable to flooding. Slope characteristics greatly influence water runoff, as steep slopes can hasten water flow during rainfall events [66]. This increases velocity results in larger volumes of water entering river systems or flowing downstream, leading to flash flooding in areas with significant gradients [67]. Additionally, steep slopes are prone to erosion during heavy rains, causing sediment accumulation downstream, which can result in water overflow into adjacent areas and elevated flood risks [68,69]. Hence slope and elevation were considered factors influencing flooding within the study area. Low-lying areas, which are often heavily populated, with high precipitation records are at greater risk of flooding [70]. Consequently, infrastructure situated at lower elevations, such as roads, agricultural lands, and buildings, are more prone to flood damage [70].

Population growth and a consequent urbanization are reckoned to have significant influence on LULCC, and pluvial flooding [2,71,72]. Urban expansion transforms vegetation into settlement areas, facilitating faster water flow in built-up areas compared to forests [2,71,72]. These factors have varying contributions to flooding, and hence the Analytical Hierarchy Process (AHP) algorithm was employed to assign weights to each of the factors i.e. precipitations, drainage density, LULC, slope, and elevation based on their influence on flooding between 0 and 1 [1,4,6]. GIS can map topographical features using elevation, and slope together with LULC data of high resolution to the highest accuracy [73]. Therefore, the combination of RS, and GIS techniques, and AHP algorithms, have been used in this study.

### 2.5 Map generation criterion

The geospatial analysis was conducted using ArcMap 10.8 software (Esri, USA). Elevation, slope, and drainage density maps were derived from a digital elevation model (DEM) data of the study area, which had a resolution of 1 arc downloaded from USGS. The DEM was imported into ArcMap 10.8 software. The “Extract by Mask” tool under the Spatial Analyst Arc Toolbox. This gave the DEM of the study area which was utilized as an input raster for the slope, drainage density, and elevations. To obtain slope, the Spatial Analyst Arc Toolbox was clicked to expand the other tools. The slope tool was selected from the Surface tool. Within the slope tool, the input raster was the DEM raster image extracted to the study area. The generated slope (in Degrees) value was reclassified into five classes.

Drainage areas were computed using a single-direction flow algorithm, based on the steepest downslope neighbor of each pixel [62]. To generate the drainage density, these procedures were followed: The Fill tool under the Hydrology tool in the Spatial Analyst Toolbox was used to fill any sinks in the DEM to ensure that the flow direction calculated was accurate. Afterward, the Flow Direction tool under the Spatial Analyst Toolbox was utilized to create a raster showing the flow direction from each cell. The input raster was the fill DEM. The Flow direction was then used as an input raster to compute the accumulated flow to each cell using the Flow Accumulation tool in ArcGIS. Moreover, the Stream Network was generated using the Flow Accumulation values as an Input raster using the Con tool under the Conditional tool. This has created a binary raster where cells with flow accumulation above the threshold were considered part of the stream network. The Stream to Feature tool under Hydrology was employed to convert the binary stream raster to a polyline feature.

(1) $$Drainage\;density\;\left(Dd\right)=\frac{Total\;Length\;of\;Streams}{Area\;of\;the\;Watershed}$$

The Line Density tool in Density under the Spatial Analyst Toolbox was employed to calculate the Dd using the relation in equation (1). The Input feature was the polyline feature with the unit being square kilometer. The DEM was reclassified into 5 classes, portraying the elevations of the study area using the Reclassify tool.

The land use and land cover (LULC) map was generated from Sentinel-2 satellite imagery obtained in 2024. The sentinel −2 imagery bands 2, 3, 4, and 8 of spatial resolution of 10 m were also imported into ArcMap 10.8 and pre-processed (i.e. mosaicked to ensure the images covered the entire study area) using the Mosaic tool. Afterward, the bands were extracted to the study area using the “Extract By Mask” tool under the Spatial Analyst Toolbox. The Composite Bands tool in the Data Management Toolbox was employed to create a single raster for the four bands. The maximum likelihood (ML) classification algorithm has proven to give highly accurate classification [74]. Hence the maximum likelihood (ML) classification algorithm within the spatial analyst toolbox was employed to classify the raster bands and produce a classified raster output using a training data signature file created from the composite raster image. An accuracy assessment of the LULC map was then performed. Studies have proven that kappa statistic values of 0.80 and above show a more accurate classification [75]. The “STRATIFIED_RANDOM” sampling strategy was used to generate 109 random points within the LULC map of the study area. Google Earth Engine (GEE) was employed to verify ground truth. Afterward, the “Compute Confusion Matrix” tool was used to analyze the accuracy. The percentage accuracy and kappa value are displayed in Table 2.

**Table 2:** LULC map accuracy assessment results for 2014 and 2024.

ClassValue	Built-up area	Forest	Cropland	Water Body	Bareland	Total (User)	User Accuracy (%)
Built-up area	21	0	1	0	1	23	91.30
Forest	0	23	1	0	0	24	95.83
Cropland	0	0	27	0	0	27	100.00
Water Body	0	0	0	10	0	10	100.00
Bareland	0	0	4	0	21	25	0.84
Total (Producer)	21	23	33	10	22	109	
Producer Accuracy (%)	100.00	100.00	81.82	100.00	95.45		
Overall Accuracy (%)							93.58
Kappa							0.918001

### 2.6 The AHP weight allocation process

The Analytic Hierarchy Process (AHP) is a mathematical method used to address complex multi-criteria decision-making processes by assigning weights to each criterion. This approach evaluates various parameters and ranks them according to a set of criteria [[76], [77], [78]]. According to Shareef et al. [74] in the initial stage, as proposed by Saaty in 1980, criteria, sub-criteria, and decision alternatives were developed in alignment with the study's objectives. For this study, five parameters that influence flooding precipitation, drainage density, LULC, elevation, and slope were considered as criterion. Consequently, the classes for each parameter such as forest, cropland, built-up area, bareland, and road networks for LULC criteria form the sub-criteria.

Furthermore, a pairwise comparison of the various parameters influencing flooding was employed by the AHP to assess the weight or rank of each parameter and its sub-classes. The Saaty comparison pairwise scale ranging from 1 to 9 was used to rate each parameter and its sub-classes against the other parameters and their sub-classes (Table 3) [77], [79], [80]. For example, as the precipitation is a direct contributor to flooding in the area, it is given a higher weight in the comparison scale than other factors. Likewise, based on its importance, drainage density was given the second-highest value after precipitation.

**Table 3:** AHP scale of pairwise comparison matrix (Source: Saaty [79]).

Intensity of importance	Definition	Explanation
**1**	Equal importance	Two activities contribute equally to the objective
**3**	Moderate importance	Experience and judgment slightly favour one activity over another
**5**	Strong importance	Experience and judgment strongly favour one activity over another
**7**	Demonstrated importance	An activity is favoured very strongly over another; its dominance demonstrated in practice
**9**	Extreme importance	The evidence favouring one activity over another is of the highest possible order of affirmation
**2-4-6-8**	Intermediate values	

The pairwise comparison matrix is represented in equation (2) as:

(2) $$M=\left(\begin{array}{c} 1\;a_{12}\;a_{13}\;\ldots \;\ldots \;a_{1n}\\ a_{21}\;1\;a_{23}\;\ldots \;\ldots \;a_{2n}\\ {\;a}_{31}\;{\;a}_{12}\;1\;\ldots \;\ldots \;\ldots \;\\ \;.\;.\;.\;.\;.\;.\;\\ \;.\;.\;.\;.\;.\;.\;\\ \;.\;.\;.\;.\;.\;.\; \end{array}\right)$$

The formula in equation (3) was then used to determine the weighting:

(3) $$a_{ij\;}=\frac{weight\;of\;attribute\;i}{weight\;of\;attribute\;j}$$

The weight values range from 1 to 9, with the matrix value of 1 if the parameters (*i and j*) have equal importance.

Following this weight assignment, the impact of each criterion on flooding was assessed. Table 4 shows the parameter weights and the effects of the pairwise comparison matrix. The weight of each sub-criteria of the key parameters was determined using the same method.

**Table 4:** The AHP pairwise comparison matrix for parameters weight.

Parameters	P	L	S	E	D	Criteria weight
**Precipitation(P)**	1	2	4	4	1	0.335
**Drainage(D)**	1	1	3	1	1	0.282
**LULC(L)**	1/2	1	5	5	1	0.214
**Elevation(E)**	1/4	1/5	1	1	1	0.098
**Slope(S)**	1/4	1/5	1	1	1/3	0.071

Finally, Table 5 shows the weights for the sub-criteria of each parameter that influences flooding (see Table 6).

**Table 5:** Weights of parameters influencing flooding.

Parameter	Sub-criteria	score
**Precipitation**	<1600	1
	1600–1640	3
	1640–1690	5
	1690–1740	7
	>1740	9
**LULC**	Built-up area	9
	Vegetation	1
	Cropland	3
	Water body	9
	Bareland	8
**Slope**	<2.04	9
	2.04–4.32	7
	4.32–7.08	5
	7.08–11.87	3
	>11.87	1
**Elevation**	<242	9
	242–264	7
	264–284	5
	284–309	3
	>309	1
**Drainage**	2.72–16.63	1
	16.63–30.53	3
	30.53–44.43	5
	44.43–58.33	7
	58.33–72.24	9

### 2.7 Accuracy assessment of AHP weighted criteria

After the various weight values for the parameters used in this study were obtained, a consistency check was required to validate the accuracy of weight values. Hence, the consistency ratio (CR) was used, which was calculated as a fraction of the matrix's consistency index (CI) on the random index (RI); and this determines the comparison's accuracy. The efficiency criteria of the AHP were estimated [76,77]. The AHP validation mathematical technique was used to assess the consistency of the paired comparisons, resulting in the calculation of a consistency ratio (CR) and consistency index (CI) using Equations (4), (5):

(4) $$CI=\frac{\lambda_{\max}-n}{n-1}$$

(5) $$CR=\frac{CI}{RI}$$

The average coherence index of the randomly generated comparison, denoted as RI (Table 6), was used alongside the highest eigenvalue of the matrix, λ_max, and the number of parameters, n, to assess consistency [78]. For the consistency ratio (CR) to be considered accurate, it must be 0.10 or below. If the CR exceeds 0.10, the pairwise comparison matrix must be recalculated [77]. The study achieved a CR value of 0.077, which is within acceptable limits, indicating that the computed weight values are accurate and consistent.

**Table 6:** Random index (RI) table (Source: Saaty [79]).

N	1	2	3	4	5	6	7	8	9	10
RI	0	0	0.58	0.90	1.12	1.24	1.32	1.41	1.45	1.49

### 2.8 Standardization of criterion and map generation

All sub-criteria were reclassified into five classes. Moreover, the AHP method scores all sub-criteria on a scale from 1 to 9, with 1 representing the least importance and 9 representing the highest importance. This standardization was essential for assessing flood vulnerability based on the level of significance of each sub-criteria (i.e. the classes for precipitation, drainage density, LULC, elevation, and slope) for the five parameters. Subsequently, the five criteria rasters of precipitation, drainage density, LULC, elevation, and slope, were overlaid, multiplying each by its respective weight assigned by AHP (Table 4) as illustrated in equation (6) and summing them together.

(6) FVI=0.335∗P+0.282∗Dd+0.214∗L+0.098∗E+0.071∗S

where:

FVI is Flood Vulnerability Index, P is precipitation, Dd is drainage density, L is LULC, E is elevation, and S is slope.

This calculated algorithm within the “Weighted Sum” tool in ArcGIS 10.8 resulted in the generation of the flood-prone areas map. The flood vulnerability map created has been reclassified into 4 classes using the Reclassify tool.

### 2.9 Validation of flood-prone area map

This study evaluated the accuracy of the flood-prone area map for the Sunyani Municipality using the error matrix method and validation data Amiri et al. [24] obtained from places where flooding has occurred and places where it hasn't based on previous studies [18]. A total of 100 flood validation sample points were employed to ascertain the accuracy of the flood-prone area map in the study area. The Create Accuracy Assessment Points tool was used to generate the location of flood zones within the flood-prone map. GEE was employed to take the coordinates of these locations, and taking the landmarks of these locations into account, an on-site visit to the places was done. The outcome reveals that the places highly flood-prone are places of high build-up areas with sparse vegetation, high drainage density, and higher precipitation. The responses from the surveys were entered and the Confusion Matrix tool in ArcMap was used to evaluate the accuracy of the flood-prone areas classification map.

## 3 Results

**Fig. 3:**
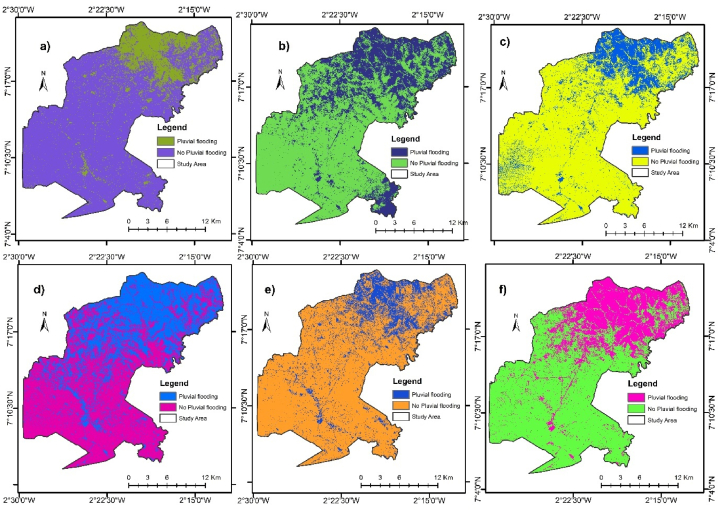
Map of pluvial flood occurrences in the study area in (a) 2014 (b) 2016 (c) 2018 (d) 2020 (e) 2022 (f) 2024.

To detect the evolution and levels of pluvial flood occurrences in the study area, historic pluvial flooding maps were generated from 2014 to 2024 on a two year interval. Fig. 3 shows the trends of occurrences of pluvial flooding in the area, from 2014 to 2024. In 2014, the spatial extents of pluvial flood occurrence was 93.86 km^2^ (18.54 %). By 2024, a 10 year period, the spatial extents of pluvial flooding has extended to 154.69 km^2^ (30.55 %). From Fig. 3, areas that did not experience flooding in 2014 have become inundated subsequently.

**Fig. 4:**
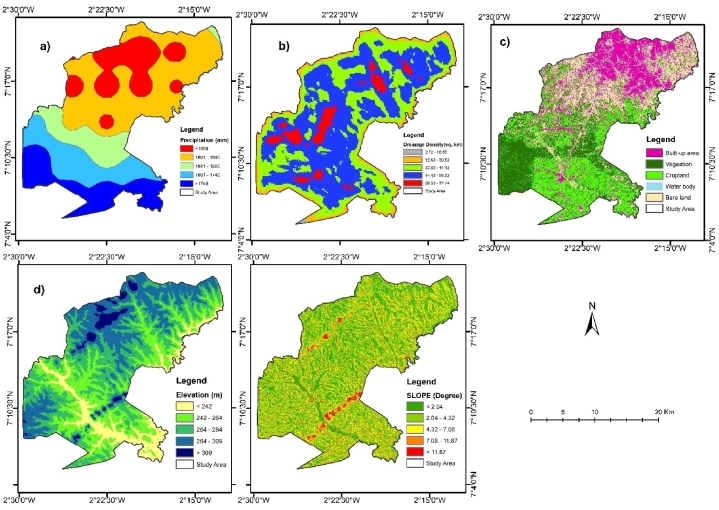
Map of biophysical factors influencing flooding (a) Precipitation (b) Drainage density (c) LULC (d) Elevation (e) Slope.

Both Table 4 and Fig. 4 illustrate the extents of prevailing biophysical factors influencing flooding in the study area. It is found that in the study area, precipitation has the highest influence on pluvial flooding, followed by drainage density.

With precipitation greater than 1600 mm, it has an elevation greater than 264 m above mean sea level. Additionally, most of the LULC class of forest and croplands are situated around drainage density greater than 44.43 km2 while the built-up area has a slope less than 4.32°. The highly flood-prone class comprises 107.96 km^2^ (21.32 %) of the study area, and this covers largely the southern and northeastern parts of the study area (Fig. 6). Built-up areas of the LULC are clustered in the northern part of the study area (Fig. 4c). Based on the study of Abdulkareem et al. [71] and Siswanto & Francés [72], conversion of forest to built-up and bareland through urbanization and other land use types makes the land more susceptible to flooding. Locations of larger drainage density are more prone to flooding than those of lower drainage density [16,17]. Thereby, the largest drainage density was between 58.33 km^2^ to 72.24 km^2^ and had a score of 9, and the least was between 2.72 km^2^ to 16.63 km^2^ and had a score of 1 on a scale of 1–9 by the AHP (Table 5).

**Fig. 5:**
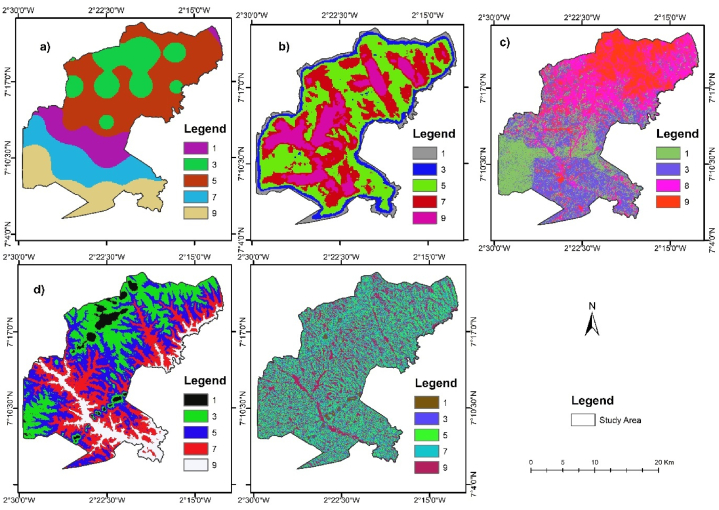
Map of standardized biophysical factor influencing flooding in the study area with 9 being highest influence and 1 lowest (a) Precipitation (b) Drainage density (c) LULC (d) Elevation (e) Slope.

The AHP was employed to assign weights to all the sub-criteria to understand the significance level of each biophysical factor influencing flooding in the study area. The class of each parameter was reclassified for standardization as displayed in Fig. 5.

**Fig. 6:**
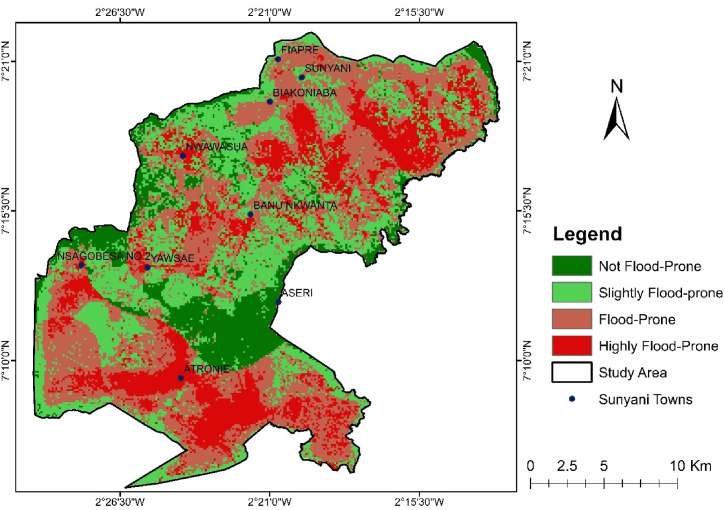
Map of the spatial distribution of flood-prone areas within the study area.

Fig. 6 display map of the spatial distribution of flood-prone areas within the municipality given the presence and significance of each of these influencing factors to pluvial flooding in the study area. 21.32 % of the study area is highly flood-prone and distributed across the southern and northern regions (Table 7). Areas that are flood prone covers 200.74 km^2^ of the study area and these locations are close to the highly flood-prone places.

**Table 7:** Flood-prone zones in square kilometers and percentages.

Zone	Area (km^2^)	Percentage (%)
Highly Flood-prone	107.96	21.32
Flood-Prone	200.74	39.65
Slightly Flood-prone	142.07	28.06
Not Flood-Prone	55.56	10.97
	506.33	100.00

## 4 Discussion

The results of this study reveal critical complex relations between land use, hydrology, and urban development. One of the most crucial discoveries made by the research is that changes in land use have a considerable influence on flooding patterns. In Fig. 3, it is observed that urbanization has been rapid from 2004 to date as a result of rapid population growth and population density. This has turned natural landscapes into built environments exposing the land to increased surface runoff rates. The LULC classes such as vegetation, cropland, built-up areas, water bodies and bare land illustrate differing influence to flooding with water bodies and built-up areas and bare land receiving the higher weights assignment by the AHP. Consequently, this emphasizes the need for sustainable urban planning with urban green spaces incorporated as a means to control pluvial flooding. The spatial distribution of pluvial flood-prone zones indicate that the areas, highly flood-prone and flood-prone are areas with higher drainage density, lower elevation, lower slope, and higher precipitation within the built-up areas in the municipality. The not flood-prone and slightly flood-prone areas are characterized by higher elevation and slope, smaller drainage density, and lower precipitation within the vegetation and cropland places.

The highly flood-prone areas are Anesim, Tonsuom estate, Mensakrom, SDA basic School, Glory Tabernacle Church, Atronie, and Atuahenekrom. The drainage density in these locations is between 44.43 km^2^ and 72.2 km^2^ (Fig. 4 and Table 5). Landmark sites such as Atronie Roman School, FEDCO Nsoatre 'B' Depot, Presbyterian Church of Ghana -Ascension Congregation, and Saint Paul Parish Church were all found within the highly flood-prone areas at Atronie. Atuahenekrom is located at the high precipitation zones of 1740 mm and low laying with elevations less than 264 m (Fig. 4). Furthermore, it has a high drainage density between 44.43 km^2^ and 58.31 km^2^. This makes this town experience floods during heavy downpours, with attestation from the residents. The next class is flood-prone areas. It covered landmark sites including Newtown, Nkwarbeng, Baakoniaba, Kootokrom, Penkwase, the University of Energy and Natural Resources (UENR), Yawhima and Bosoma Market, Sunyani Airport, Chiribija, Twene Amanfo Senior High School, Sacred Heart School, and Sunyani Teaching Hospital and Divine Montessori JHS. These locations are residential and public places full of infrastructure. Moreover, they are low laying with elevations less than 264 m, with drainage density between 30.53 km^2^ and 58.33 km^2^. These results confirm the findings of a study Wanyor & Morkla [18] conducted that revealed the number of essential facilities identified within the risk zones were schools and religious facilities.

The implications of this are loss of property, lives and economic activities to the country should there be an intense rainfall, holding all other observed influencing factors constant. The schools and churches mentioned have 100s of people within those facilities at any time. During the ground truthing, residents in these locations attested to flash floods during high precipitation, confirming the accuracy of our flood-prone areas model. Previous studies indicate that urbanization is linked with increased flood risks. Examples include the works of Twum & Abubakari's [11], Rosenzweig et al. [6] Alhassan [14] and Barbier [81]. The integration of slope characteristics into the analysis further enriches the understanding of flood susceptibility. As explained by Rudra & Sarkar [66], the influence of slope on water runoff is critical in determining flood risks. The study's findings suggest that areas with steep slopes may experience rapid runoff, exacerbating flooding in downstream regions in the study area. This relationship underscores the importance of considering topographical features in flood risk assessments, as they significantly influence hydrological responses to rainfall events [51].

Furthermore, the development of unregulated physical structures in waterways can partly be attributed to the cause of increased incidences of flooding in the Sunyani Municipality as the population is always on the ascendency. Also, through the ground truthing, it has been observed that most drains are choked with loads of sediments. This has significance for surface runoff and pluvial flooding, which occurred in June 2019. The June 2019 pluvial flooding led to the destruction of most office and household items in the Sunyani Municipality [18]. The significance of flood risk and flood hazard maps, therefore, cannot be underestimated in the Sunyani East Municipality areas that have experienced previous events. They help detect areas at risk of flooding disasters, indicate looming flooding, subject to technical interpretation, provide information on the spatial extent, depth, and flood frequency of a region. However, in a matter of interpretation, the specification and ranking of flood-prone regions based on flood risk maps is a function of the end users’ requirement [82].

In Ghana, the end-users of flood risk and flood hazard maps include but are not limited to National Disaster Management Organization (NADMO), the Environmental Protection Agency (EPA), Metropolitan, Municipal, and District Assemblies (MMDAs), Ministry of Food and Agriculture, local communities, and Non-Governmental Organizations, and design engineers. Urban Spatial Planners at the MMDAs may use these maps as important tools in limiting the extent of damage caused by flood hazards through redesign of land use and infrastructure. This will further help reduce mortalities due to flood hazards, help in evacuation plans, and assist in raising awareness [83]. In particular, robust forecasting of a trigger rainfall together with a method for identifying locations that are susceptible to pluvial flooding have been recommended in literature and in practice to responding organizations. At the moment, the Sunyani Municipality does not have such a mechanism in place for prioritized actions.

It is, therefore, recommended that the flood hazard risk map be used to instigate the adoption of a risk-based tiered approach such that prioritized actions can be effectively targeted on areas that require further detailed assessment. This may further require the use of surface flow modelling or integrated urban flood modelling. Site inspections of locations indicated as having a higher level of flood hazard risk (Fig. 3) will provide valuable verification of the mapping, understanding of locally significant issues, and identification of possible mitigation measures. Measures may include the adoption of residential flood insurance schemes covers for property owners. Looking into the future, it is recommended that relevant partnership should be established between the responding organizations, weather forecasting institutions for extreme rainfall alert services; and scientific institutions for near real-time mapping of potentially vulnerable areas to underpin an effective response. Most critical is an improved awareness of areas that may be more vulnerable to pluvial flooding in the municipality. With such a partnership, emergency planning and response can be undertaken on a proactive basis rather than current reactive-base responses due to dearth of intelligence. Once vulnerable areas have been identified, ready emergency response itinerary can be installed at key locations to mitigate or manage pluvial flooding.

## 5 Conclusion

In this study, five parameters which are precipitation, drainage density, LULC, elevation, and slope that influence flooding were utilized to map flood-prone areas within the Sunyani Municipality. These parameters were integrated in GIS and using an AHP, weights were assigned to each parameter based on the level of influence on flooding. The findings reveal that 21.32 % of the Sunyani Municipality lies highly flood-prone area, 39.65 % in a flood-prone area, while 28.06 % and 10.97 % in slightly flood-prone and not flood-prone areas respectively. Built-up areas close to watersheds with lower elevations and larger drainage density are the places highly flood-prone. Given this finding, the government and relevant stakeholders in disaster risk management are better informed to effectively plan and prevent flood challenges in the Sunyani Municipality. Moreover, urban spatial planners in the study setting can consider incorporating flood hazard maps into their spatial plans for proactive physical developments. Furthermore, further studies can include other parameters to model flood in the study setting. This study provides an effective preliminary indication over a wide area of the level of pluvial flood hazard, and the extent of risk. The findings can help to inform Preliminary Flood Risk Assessments and the development of a resource efficient strategy for subsequent mapping.

## Data availability statement

The geospatial layers developed in the frame of the study using satellite imagery, AHP, ArcGIS, and the results of the study for flood risk mapping can be accessed from our Google drive. Access to the drive shall be made available upon request.

## Funding

This research has been funded by the Group on Earth Observation flagship Land Degradation Neutrality (GEO-LDN) Secretariat hosted under the German International Cooperation for Development (GIZ), Bonn, Germany.

## CRediT authorship contribution statement

**Aaron Tettey Tetteh:** Writing – review & editing, Writing – original draft, Visualization, Validation, Software, Resources, Project administration, Methodology, Investigation, Formal analysis, Data curation, Conceptualization. **Abdul-Wadood Moomen:** Writing – review & editing, Writing – original draft, Visualization, Validation, Supervision, Software, Resources, Project administration, Methodology, Investigation, Formal analysis, Data curation, Conceptualization, Funding acquisition. **Lily Lisa Yevugah:** Writing – review & editing, Writing – original draft, Visualization, Validation, Supervision, Software, Project administration, Methodology, Investigation, Formal analysis, Data curation, Conceptualization. **Albert Tengnibuor:** Writing – review & editing, Writing – original draft, Visualization, Validation, Supervision, Software, Methodology, Investigation, Formal analysis, Data curation, Conceptualization.

## Declaration of competing interest

The authors declare that they have no known competing financial interests or personal relationships that could have appeared to influence the work reported in this paper.
